# Strangulated Small Bowel Obstruction Caused by a Hernia-Related Adhesive Band after Successful Reduction of an Incarcerated Inguinal Hernia

**DOI:** 10.70352/scrj.cr.26-0559

**Published:** 2026-09-10

**Authors:** Kazuya Takabatake, Keigo Hanya, Ryo Ishida, Yudai Nakabayashi, Tomoki Konishi, Toru Mizutani, Yoshitaka Nakamura, Susumu Nakashima, Manabu Takemura, Hiroshi Koike, Fumihiro Taniguchi, Atsushi Shiozaki

**Affiliations:** 1 Department of Surgery, Japanese Red Cross Kyoto Daini Hospital, Kyoto, Kyoto, Japan; 2 Division of Digestive Surgery, Department of Surgery, Kyoto Prefectural University of Medicine, Kyoto, Kyoto, Japan

**Keywords:** adhesions, inguinal hernia, intestinal obstruction, intestinal strangulation, reduction en masse

## Abstract

**INTRODUCTION:**

Incarcerated inguinal hernia is a common surgical emergency. Persistent bowel obstruction after successful reduction is typically attributed to reduction en masse, whereas other mechanisms of ongoing strangulation are rarely reported. We describe a rare case in which bowel strangulation persisted despite successful reduction of an incarcerated inguinal hernia due to a hernia-related adhesive band.

**CASE PRESENTATION:**

A 92-year-old man presented with an incarcerated left inguinal hernia. Manual reduction was successfully performed, and contrast-enhanced CT confirmed reduction of the herniated bowel into the abdominal cavity without obvious signs of ischemia or persistent incarceration. However, abdominal pain and small bowel obstruction persisted. Because ongoing strangulation could not be excluded, emergency laparoscopic exploration was undertaken. Intraoperatively, the incarcerated bowel had been completely reduced into the abdominal cavity, and no evidence of reduction en masse was identified. Instead, a fibrous band extending from the sigmoid colon to the retroperitoneum formed a constricting ring that entrapped a segment of small bowel, resulting in strangulated obstruction. Laparoscopic division of the band immediately relieved the strangulation, and bowel resection was avoided. Retrospective review of the post-reduction CT revealed subtle findings consistent with a closed-loop obstruction.

**CONCLUSIONS:**

This case highlights that successful reduction of an incarcerated inguinal hernia does not necessarily indicate resolution of strangulation. Persistent symptoms after reduction should prompt consideration of ongoing bowel strangulation even when CT findings appear reassuring.

## Abbreviations

CRP
: C-reactive protein
TAPP
: transabdominal preperitoneal

## INTRODUCTION

Incarcerated inguinal hernia is a common surgical emergency. Manual reduction is often attempted when bowel viability is preserved and signs of strangulation are absent. However, persistent abdominal pain or bowel obstruction after successful reduction warrants careful evaluation. Reduction en masse, in which the hernia sac and incarcerated bowel are displaced into the preperitoneal space while obstruction persists, is a well-recognized complication after reduction of an incarcerated inguinal hernia.^1,2)^ In contrast, other mechanisms of ongoing strangulation following successful reduction are rarely reported. We report a rare case of strangulated small bowel obstruction caused by a hernia-related adhesive band after successful reduction of an incarcerated inguinal hernia.

## CASE PRESENTATION

**Fig. 1:**
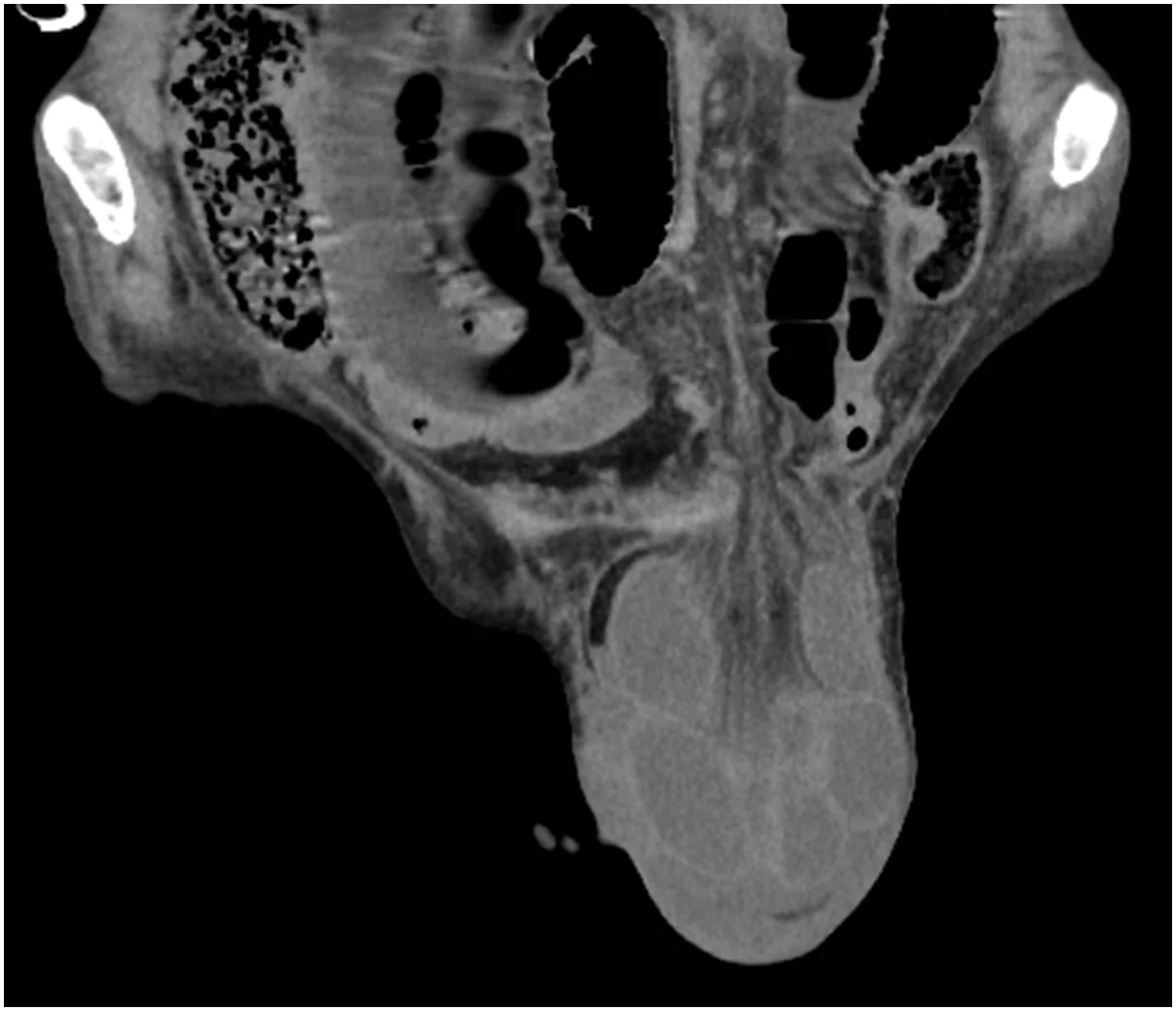
Pre-reduction CT imaging. Pre-reduction CT showing an incarcerated left inguinal hernia containing small bowel.

A 92-year-old man presented to the emergency department of the Japanese Red Cross Kyoto Daini Hospital with acute left groin swelling and abdominal pain. He had been aware of a left inguinal hernia for approximately 20 years. Because of his advanced age, conservative management had been recommended at another hospital. Although the exact frequency could not be determined, he had experienced repeated episodes of successful self-reduction over the years, all of which had been followed by prompt symptom resolution. He had never previously experienced persistent abdominal pain or bowel obstruction after self-reduction. On physical examination, a tender, irreducible bulge was noted in the left inguinal region. Initial laboratory tests revealed a white blood cell count of 7400/μL, CRP of <0.09 mg/dL, and serum lactate of 1.6 mmol/L. CT demonstrated a left incarcerated inguinal hernia containing small bowel (**Fig. 1**).

**Fig. 2:**
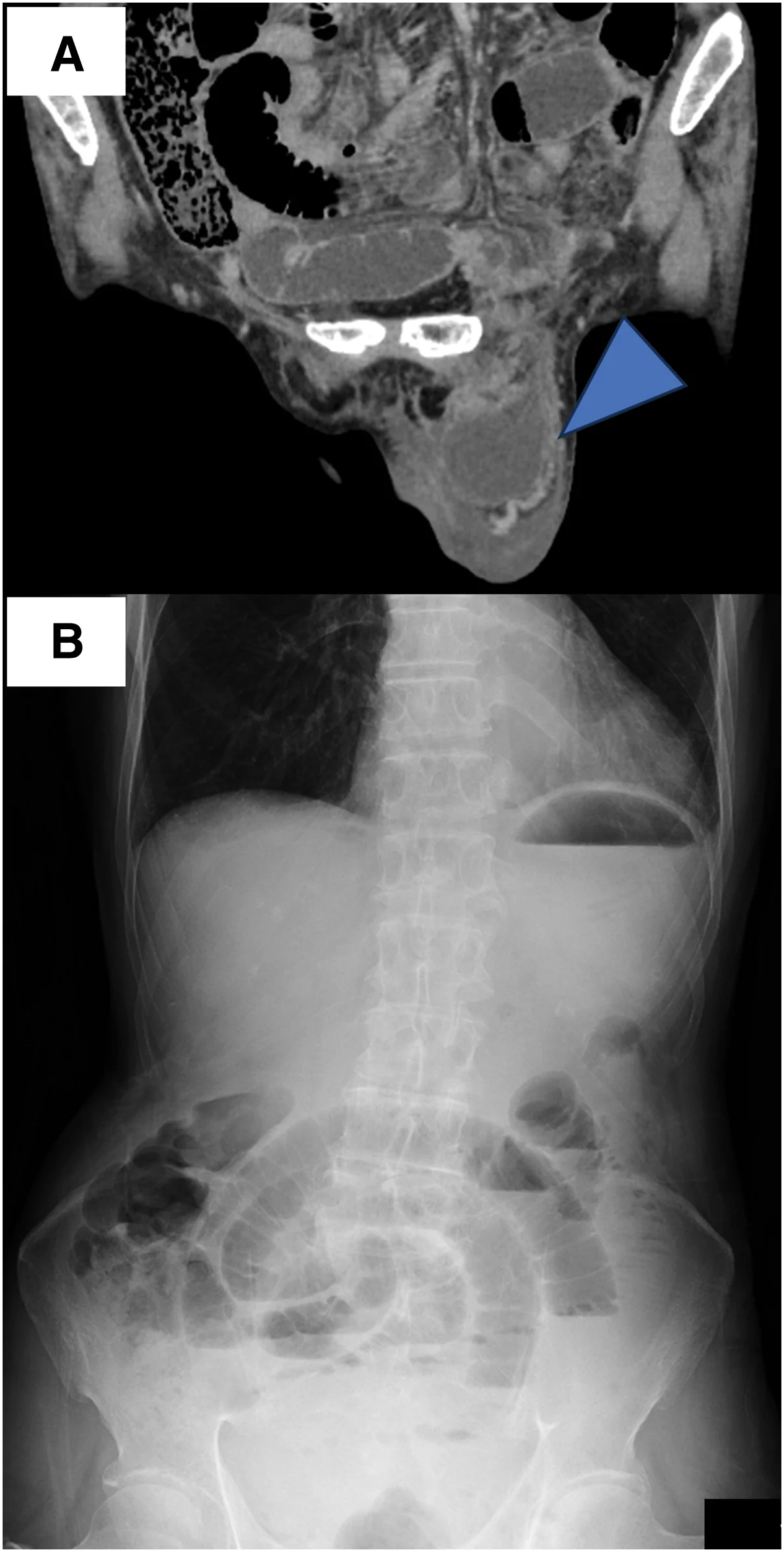
Post-reduction imaging. (**A**) Contrast-enhanced CT obtained immediately after reduction demonstrating reduction of the herniated bowel without obvious signs of ischemia or persistent incarceration. The residual hernia sac contained only ascites (arrowhead). (**B**) Abdominal radiograph obtained approximately 8 hours after reduction showing the persistent dilatation of the small bowel with multiple air-fluid levels.

Manual reduction was successfully performed after admission. Subsequent contrast-enhanced CT confirmed reduction of the herniated bowel into the abdominal cavity (**Fig. 2A**). Bowel wall enhancement was preserved, and no obvious signs of ischemia, perforation, or persistent incarceration were identified. Therefore, the patient was admitted for observation because of persistent mild abdominal pain. Approximately 8 hours after reduction, abdominal radiography continued to demonstrate small bowel dilatation with multiple air-fluid levels (**Fig. 2B**). Throughout the observation period, the patient continued to complain of mild abdominal pain. Physical examination revealed persistent tenderness from the epigastric to the periumbilical region, without guarding or rebound tenderness. His vital signs remained stable, with a blood pressure of 144–163/74–80 mmHg, heart rate of 63–65 beats/min, respiratory rate of 12–15 breaths/min, oxygen saturation of 95%–96% on room air, and body temperature of 35.7°C–37.5°C. Laboratory tests, including the white blood cell count, CRP, and serum lactate, were not repeated during this period. Despite the absence of clinical deterioration or peritoneal signs, the persistent abdominal pain and unresolved small bowel obstruction raised concern for ongoing strangulation. Therefore, approximately 12 hours after manual reduction, emergency laparoscopic exploration was performed.

**Fig. 3:**
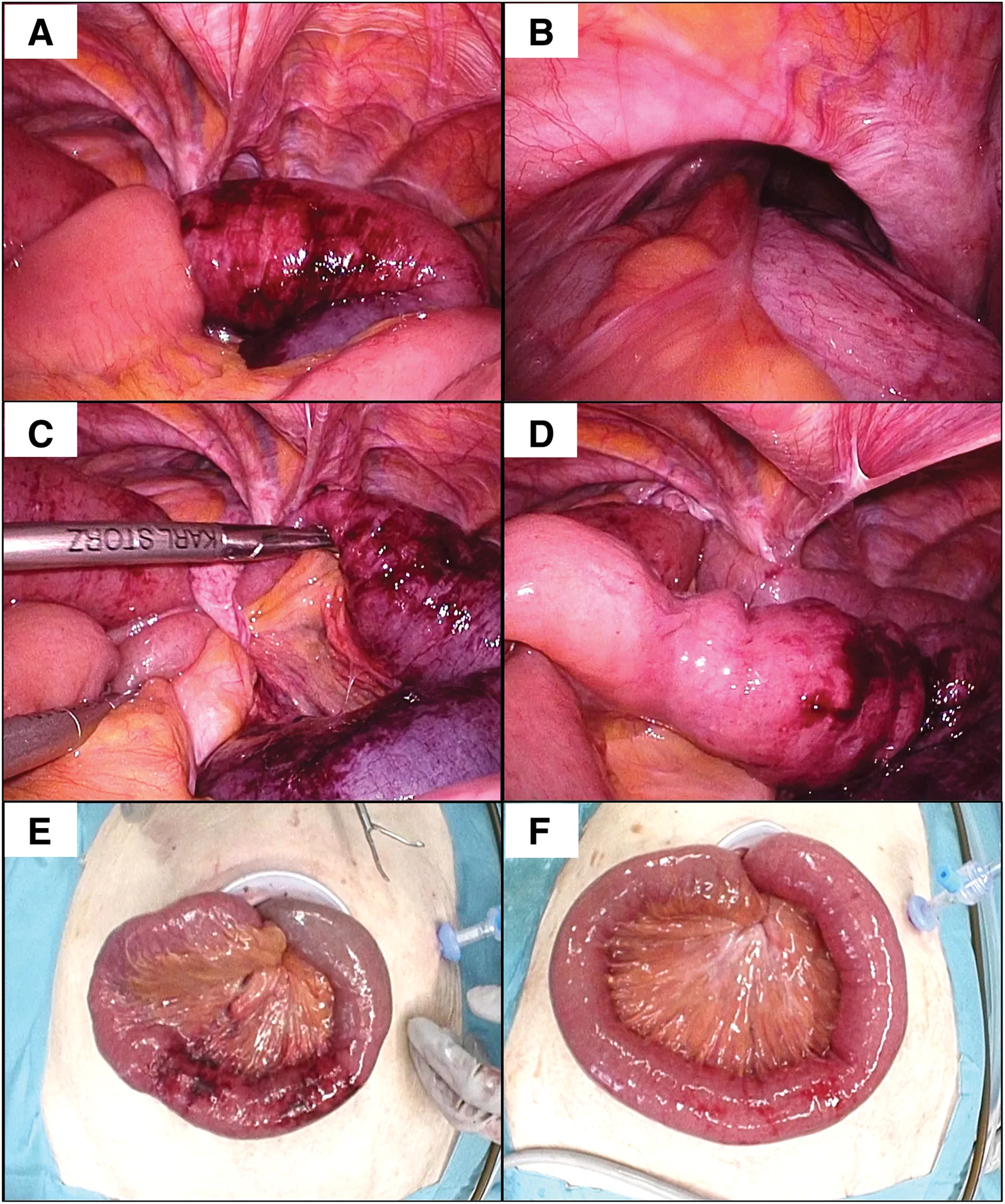
Intraoperative findings. (**A**) Congestion of a segment of the small bowel without evidence of reduction en masse; (**B**) adhesions involving the hernia sac and the herniated sigmoid colon; (**C**) a fibrous band extending from the sigmoid colon to the retroperitoneum, forming a constricting ring that entrapped a segment of small bowel and resulted in strangulated obstruction; (**D**) immediate relief of the strangulation following laparoscopic division of the fibrous band; (**E**) congested small bowel immediately after release of the strangulation; and (**F**) recovery of bowel color and resolution of congestion after release.

Laparoscopic examination revealed no evidence of reduction en masse, but congestion of a segment of the small bowel was noted (**Fig. 3A**). The incarcerated bowel had been completely reduced into the abdominal cavity. Dense adhesions involving the hernia sac and the herniated sigmoid colon were observed (**Fig. 3B**). A fibrous band extending from the sigmoid colon to the retroperitoneum formed a constricting ring, beneath which a segment of small bowel had become entrapped, resulting in strangulated obstruction (**Fig. 3C**). The fibrous band was divided laparoscopically, immediately relieving the strangulation (**Fig. 3D**). Following release, bowel color and peristalsis rapidly improved, and bowel resection was deemed unnecessary (**Fig. 3E** and **3F**). Because the hernia defect was considered the underlying cause of the pathological process and the patient had a symptomatic inguinal hernia, TAPP repair was subsequently performed during the same operation.

The postoperative course was uneventful, and the patient was discharged on POD 5. No recurrence of bowel obstruction or inguinal hernia was observed during follow-up.

## DISCUSSION

When symptoms fail to resolve after reduction, surgeons are generally alerted to the possibility of reduction en masse, a condition in which the hernia sac and incarcerated bowel are displaced into the preperitoneal space while obstruction persists. Because delayed diagnosis may result in bowel ischemia and necrosis, reduction en masse is widely recognized as a surgical emergency.^1,2)^ The present case differed mechanistically from reduction en masse (**Table 1**). Laparoscopic exploration demonstrated complete reduction of the incarcerated bowel into the abdominal cavity, and no evidence of preperitoneal incarceration was identified. Instead, strangulation was caused by a fibrous adhesive band associated with chronic changes around the hernia sac. Thus, although the clinical presentation closely mimicked reduction en masse, the underlying mechanism was entirely different.

**Table 1:** Comparison between the present case and reduction en masse

Feature	Present case	Reduction en masse
Herniated bowel after reduction	Completely reduced into the abdominal cavity	Remains incarcerated within the hernia sac
Site of obstruction	Intraperitoneal adhesion band	Preperitoneal hernia sac
Mechanism	Sigmoid colon–retroperitoneum fibrous band	Persistent incarceration
CT findings	Closed-loop obstruction with preserved enhancement	Persistent obstruction within the hernia sac
Surgical findings	No preperitoneal incarceration	Incarcerated bowel within the hernia sac
Treatment	Band division and hernia repair	Hernia reduction and repair

Nachimuthu et al. reported bowel strangulation caused by an omental adhesion band within an inguinal hernia sac.^3)^ However, strangulation in that case occurred within the hernia itself. In contrast, the present case involved a fibrous band extending between the sigmoid colon and the retroperitoneum, and the incarcerated bowel had already been completely reduced into the abdominal cavity. Thus, the hernia defect itself was no longer responsible for bowel obstruction after reduction; rather, a secondary adhesive band generated by chronic hernia-related inflammatory changes became the actual cause of persistent strangulation. To the best of our knowledge, this is the first reported case of persistent strangulation caused by a hernia-related adhesive band after successful reduction of an incarcerated inguinal hernia.

**Fig. 4:**
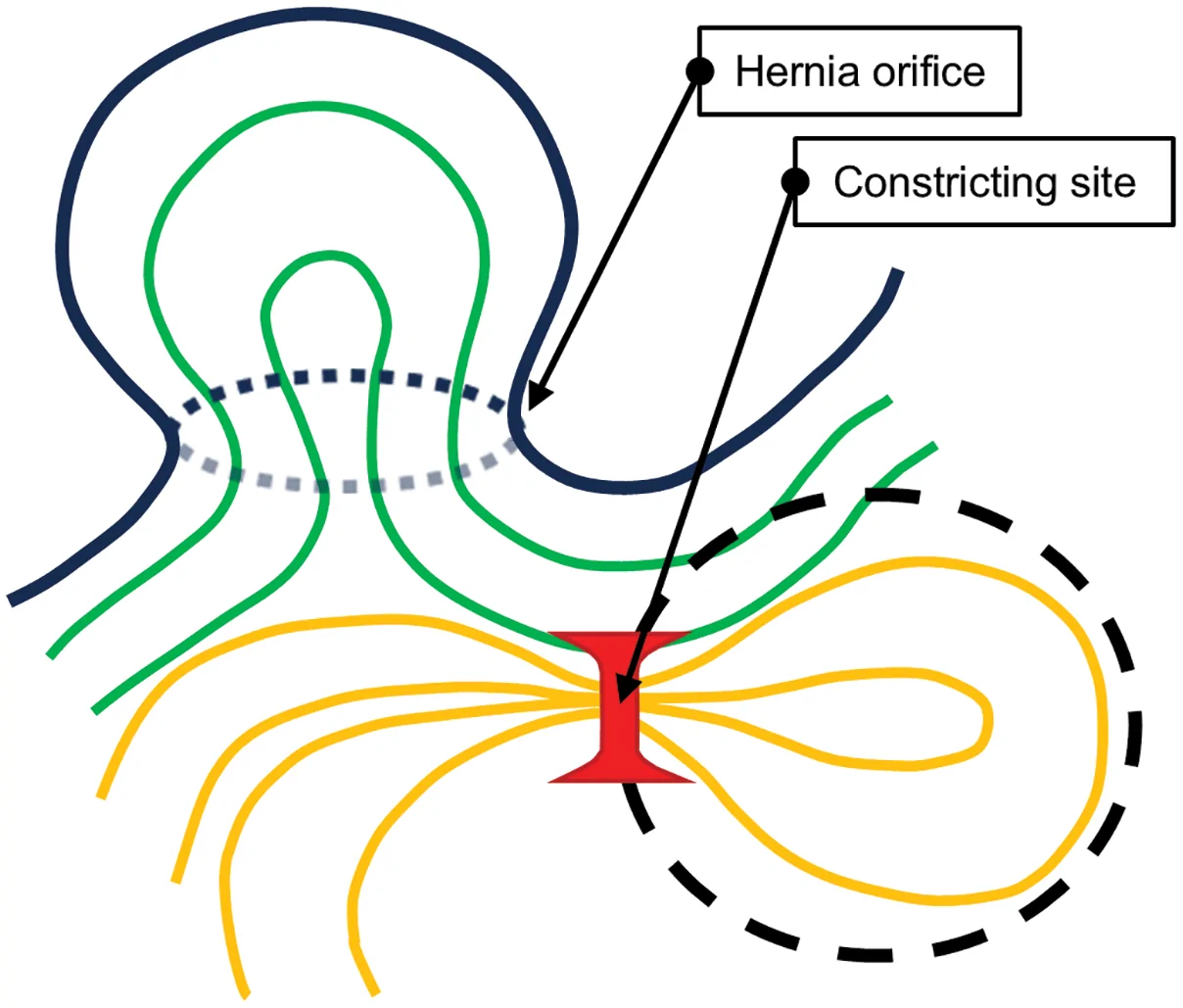
Schematic illustration of the intraoperative anatomical relationships. The blue line represents the abdominal wall and the hernia sac; the green lines, the sigmoid colon; the yellow lines, the small bowel; and the red band, the adhesive band extending between the sigmoid colon and the retroperitoneum. The dashed outline delineates the closed-loop segment. The dotted line indicates the hernia orifice, and the adhesive band entrapped the small bowel beneath it, resulting in a closed-loop strangulated obstruction.

The mechanism of band formation remains speculative. The patient had a longstanding inguinal hernia for approximately 20 years, with multiple episodes of incarceration and self-reduction. Intraoperatively, the sigmoid colon was found to have herniated into the sac and to be densely adherent to it. These repeated episodes may have induced chronic mechanical irritation and inflammation around the hernia sac, promoting adhesion formation between the hernia sac, sigmoid colon, and retroperitoneum.^4,5)^ These adhesions may have evolved into a fibrous adhesive band extending from the sigmoid colon to the retroperitoneum, creating a potential site for small bowel entrapment. The intraoperative anatomical relationships are illustrated schematically in **Fig. 4**. The present case suggests that chronic inflammatory changes associated with longstanding inguinal hernias may occasionally generate secondary causes of strangulated bowel obstruction independent of the hernia defect itself.

**Fig. 5:**
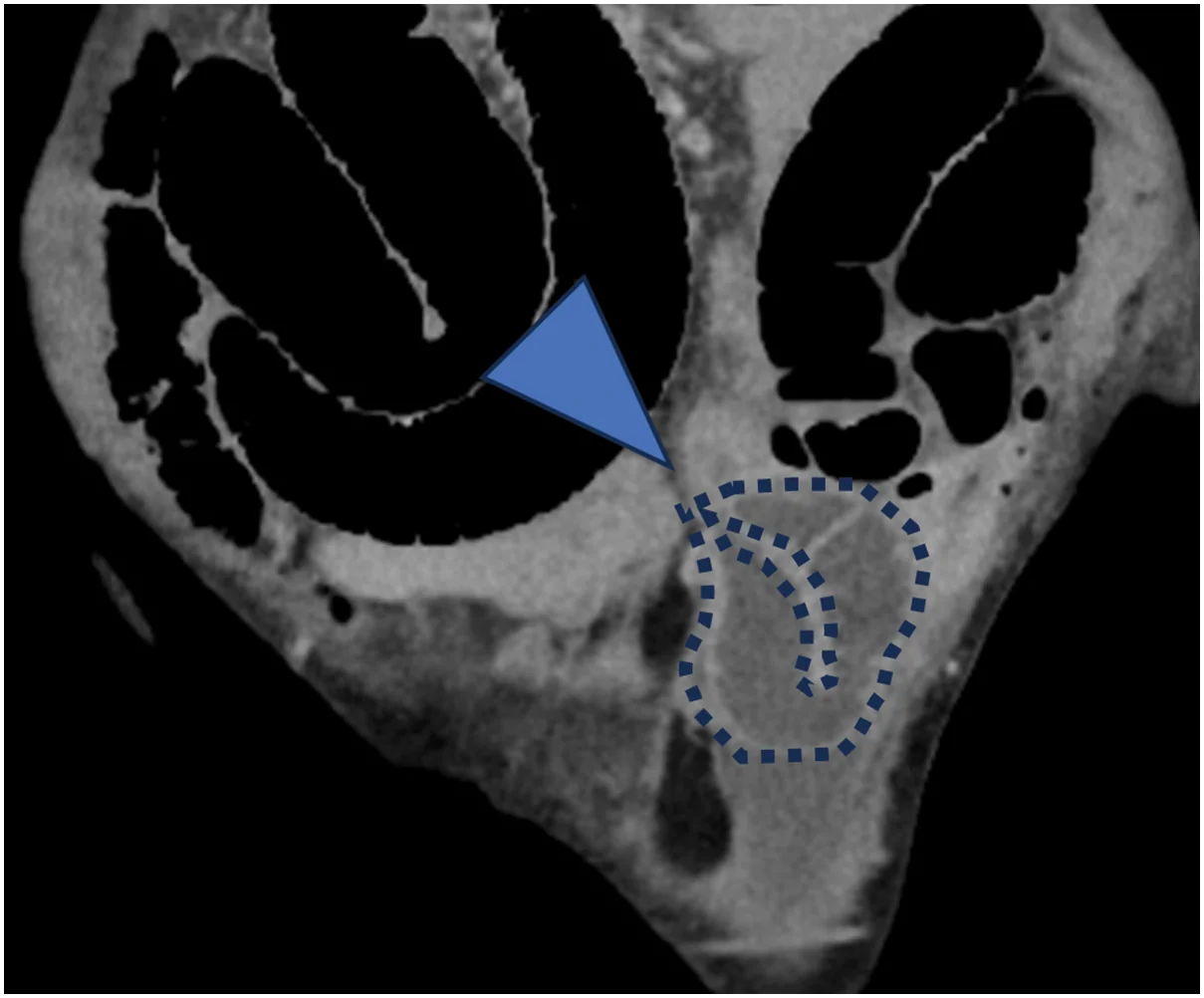
Retrospective review of post-reduction contrast-enhanced CT imaging. Retrospective review of the post-reduction CT demonstrating subtle findings consistent with a closed-loop obstruction. Dotted lines outline the closed-loop segment, while the arrowhead indicates the constricting site (transition point). Bowel wall enhancement remained preserved, and no definitive signs of ischemia were identified.

This case also highlights an important diagnostic pitfall. Contrast-enhanced CT performed after reduction demonstrated preserved bowel wall enhancement and no obvious evidence of ongoing incarceration. However, retrospective review revealed subtle findings consistent with a closed-loop obstruction (**Fig. 5**). Generalized bowel dilatation and preserved enhancement obscured the diagnosis. As demonstrated in this case, the absence of definitive ischemic findings on CT does not exclude early strangulation.^6)^

The most important clinical message is that persistent abdominal pain or unresolved small bowel obstruction after successful hernia reduction should not automatically be attributed to transient bowel edema or paralytic ileus. Although reduction en masse is the classic explanation for persistent symptoms after hernia reduction, surgeons should recognize that successful reduction does not necessarily indicate resolution of strangulation. When symptoms persist despite apparently successful reduction and reassuring CT findings, a low threshold for surgical exploration is warranted.^7,8)^ Early laparoscopic exploration may be both diagnostic and therapeutic in such situations.

## CONCLUSIONS

We report a rare case of persistent strangulated small bowel obstruction caused by a hernia-related adhesive band after successful reduction of an incarcerated inguinal hernia. Unlike reduction en masse, strangulation persisted despite complete reduction of the incarcerated bowel. Persistent symptoms after hernia reduction should prompt consideration of ongoing strangulation even in the absence of definitive ischemic findings on CT.

## DECLARATIONS

### Availability of data and materials

Not applicable. This article is a case report, and no datasets were generated or analyzed during the current study.
